# Lack of association between MTHFR Ala222Val and Glu429Ala polymorphisms and bladder cancer risk: A meta-analysis of case-control studies

**DOI:** 10.3892/br.2014.258

**Published:** 2014-03-19

**Authors:** RONG SHI, ZHEN ZHAO, HUI ZHOU, JUEYU ZHOU, WANLONG TAN

**Affiliations:** 1 Institute of Genetic Engineering, Nanfang Hospital, Southern Medical University, Guangzhou, Guangdong 510515, P.R. China; 2 Department of Urinary Surgery, Nanfang Hospital, Southern Medical University, Guangzhou, Guangdong 510515, P.R. China

**Keywords:** methylenetetrahydrofolate reductase, bladder cancer, polymorphism, meta-analysis

## Abstract

Bladder cancer is a commom malignancy in the urinary tract that is influenced by genetic and environmental factors. The role of functional polymorphisms in the methylenetetrahydrofolate reductase (MTHFR) gene with bladder cancer risk remains to be determined. This meta-analysis was performed to derive a more precise estimation of MTHFR Ala222Val and Glu429Ala polymorphisms and bladder cancer risk. Data were collected with the last report up to September 2013. A total of 3,463 cases and 3,927 controls for Ala222Val, and 3,177 cases and 3,502 controls for Glu429Ala were analyzed. The pooled odds ratios (ORs) and 95% confidence interval (CI) were estimated for the association with bladder cancer risk. Overall, no significant associations of Ala222Val and Glu429Ala polymorphisms with bladder cancer risk were found (for Ala222Val: Val/Val vs. Ala/Ala: OR, 1.02; 95% CI: 0.80–1.29; Val/Ala vs. Ala/Ala: OR, 1.02; 95% CI: 0.92–1.12; dominant model: OR, 1.01; 95% CI: 0.87–1.17; recessive model: OR, 1.00; 95% CI: 0.87–1.15; and for Glu429Ala: Ala/Ala vs. Glu/Glu: OR, 1.11; 95% CI: 0.78–1.58; Ala/Glu vs. Glu/Glu: OR, 1.16; 95% CI: 0.95–1.40; dominant model: OR, 1.15; 95% CI: 0.94–1.41; recessive model: OR, 0.96; 95% CI: 0.79–1.15). In stratified analyses by ethnicity, significant associations were observed for Glu429Ala polymorphism in individuals of Middle Eastern descent (Ala/Glu vs. Glu/Glu: OR, 2.11; 95% CI: 1.26–3.53; dominant model: OR, 2.16; 95% CI: 1.16–4.01; recessive model: OR, 1.82; 95% CI: 1.11–3.01). This meta-analysis demonstrated that overall there was no association of MTHFR Ala222Val and Glu429Ala polymorphisms with bladder cancer risk. However, in the stratified analysis by ethnicity the MTHFR Glu429Ala polymorphism was significantly associated with increased bladder cancer risk in individuals of Middle Eastern descent.

## Introduction

Bladder cancer is the most common malignant tumor of the urinary tract, with an estimated 386,300 new cases and 150,200 mortalities from bladder cancer annually (1), ranking seventh in men and seventeenth in women worldwide (2). The etiology of bladder cancer involves the interaction between genetic and environmental factors. Common risk factors include cigarette smoking, occupational exposure to aromatic amines and polycyclic aromatic hydrocarbons, inflammation of the urinary tract and consumption of certain drugs (3). Current evidence supports that molecular alterations and DNA polymorphisms potentially alter individual susceptibility to bladder cancer (4–5).

Of all the genetic susceptibility factors of bladder cancer, folate metabolic pathway has received increasing attention (5–7). Since it is the one-carbon groups donor in DNA methylation and synthesis, folate deficiency may induce DNA hypomethylation and potentially induce a proto-oncogene expression leading to cancer.

Methylenetetrahydrofolate reductase (MTHFR) is the key enzyme involved in folate metabolism, which acts as a critical metabolic node in the regulation of methylation reactions. It catalyzes the conversion of 5,10-methylenetetrahydrofolate to 5-methyltetrahydrofolate. The 5,10-methylenetetrahydrofolate is the methyl donor for *de novo* thymidine synthesis and the 5-methyltetrahydrofolate is used as a cosubstrate to convert homocysteine into methionine, which is the immediate precursor of S-adenosylmethionine and the primary methyl donor of DNA methylation. A less active form of MTHFR may result in hypomethylation, which is a candidate mechanism for the development of cancer (8).

The functional polymorphisms in the MTHFR gene, Ala222Val (C677T) and Glu429Ala (A1298C), have received increasing attention. Ala222Val homozygotes have been associated with reduced enzyme activity of ~30% of the control value (9), while the Glu429Ala homozygotes exhibit ~60% of the control activity. Heterozygotes for the Ala222Val and Glu429Ala mutations had ~50–60% of control activity (10). Since the two variants result in reduced activity of MTHFR, their associations with the susceptibility of a variety of cancers has been evaluated (11–14).

MTHFR Ala222Val and Glu429Ala polymorphisms have also been evaluated in relation to bladder cancer risk. However, the results of previous studies have yielded conflicting results. Given the amount of accumulated data, this meta-analysis was performed to derive a more precise estimation of the association of MTHFR Ala222Val and Glu429Ala and bladder cancer risk.

## Materials and methods

### Publication search

An exhaustive search of the literature was performed using the electronic databases: PubMed, EBSCO-Medline, Elsevier ScienceDirect and BIOSIS Previews for relevant articles published (up to September 2013). The articles were identified by using the search terms ‘methylenetetrahydrofolate reductase’, ‘MTHFR’, ‘NADPH2’, ‘urinary bladder’ and ‘bladder’. The search was limited to human studies with no language restrictions being applied. Additional studies were obtained through the references cited in retrieved articles on the association between the MTHFR Ala222Val and Glu429Ala polymorphisms and bladder cancer. The searching of the electronic databases and reviewing of the references in retrieved articles were independently achieved by two investigators.

### Inclusion and exclusion criteria

Inclusion criteria for the studies were: case-control studies on the association between MTHFR Ala222Val and Glu429Ala polymorphisms and bladder cancer risk, and the data of each study was required to be sufficient for statistical analysis of odds ratio (OR) and 95% confidence interval (CI). Studies that had duplicated data and in which genotypes could not be ascertained were excluded.

### Data abstraction

Information was extracted from all the eligible publications independently by two investigators as per the inclusion and exclusion criteria. For disagreements, consensus was reached by discussion of the two investigators. The data abstracted from each study were as follows: first author’s name, publication date, country, ethnicity, source of controls, number of cases and controls, and number of cases and controls for MTHFR Ala222Val and Glu429Ala polymorphisms. Individuals of different descents were classified as Chinese, European, American and Middle Eastern.

### Statistical analysis

ORs with 95% CIs were applied to evaluate the strength of association between MTHFR Ala222Val and Glu429Ala polymorphisms and bladder cancer risk. The pooled ORs were estimated for the co-dominant model (Val/Val vs. Ala/Ala and Val/Ala vs. Ala/Ala for Ala222Val; Ala/Ala vs. Glu/Glu and Ala/Glu vs. Glu/Glu for Glu429Ala), dominant model (Val/Val + Val/Ala vs. Ala/Ala for Ala222Val; Ala/Ala + Ala/Glu vs. Glu/Glu for Glu429Ala) and recessive model (Val/Val vs. Val/Ala + Ala/Ala for Ala222Val; Ala/Ala vs. Ala/Glu + Glu/Glu for Glu429Ala), respectively. The Chi-square test-based Q-statistic (Q test) was applied to assess heterogeneity among the studies. The fixed-effects model was used to calculate the pooled ORs if no heterogeneity was detected (Ph≥0.05 by Q test) (15). Otherwise, the random-effects model was applied (Ph<0.05 by Q test) (16).

Subgroup analyses were performed by ethnicity and source of controls. The sensitivity analyses were conducted by excluding each study at a time to determine its effect on the overall estimation, since all the studies indicated that they conformed to the Hardy-Weinberg equilibrium (HWE). The publication bias was estimated by the funnel plot, in which the standard error of log (OR) was plotted against its log (OR) for each study. Egger’s linear regression test was applied to assess the asymmetry of the funnel plot, with P<0.05 indicating an asymmetric plot and a possible publication bias (17). In this meta-analysis, the Stata version 11.0 (StataCorp, College Station, TX, USA) was applied for all the statistical tests.

## Results

### Flow of included studies

A total of 420 articles potentially relevant to the searching terms were screened, including PubMed, 25; EBSCO-Medline, 24; Elsevier ScienceDirect, 338; and BIOSIS Previews, 33. Based on the inclusion criteria, a total of 13 studies (5–7,18–27) with full-text articles on polymorphisms of MTHFR Ala222Val and Glu429Ala and bladder cancer risk were identified as eligible. Of the 13 studies, two studies (22,24) were excluded due to duplicated data with the study by Rouissi *et al* (6).

### Study characteristics

A total of 11 studies (5–7,18–21,23,25–27) were included in this meta-analysis for Ala222Val (including 3,463 cases and 3,.927 controls) and 9 studies (5–7,19–21,23,25,27) were included for Glu429Ala (including 3,177 cases and 3,502 controls) (Table I). Three of the 11 studies were population-based and eight were hospital-based studies. The studies indicated that the distribution of genotypes in controls was consistent with HWE. Detailed data from the included studies were abstracted (Table II).

### Quantitative data synthesis

**Figure 1:**
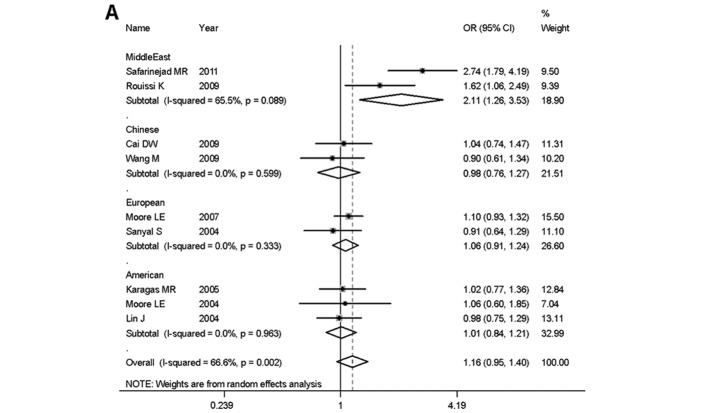

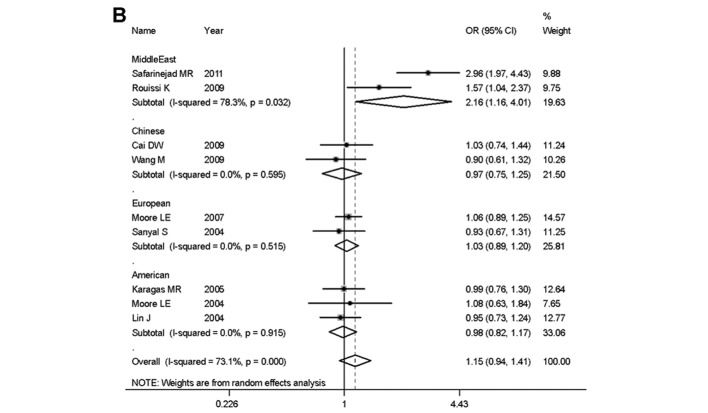

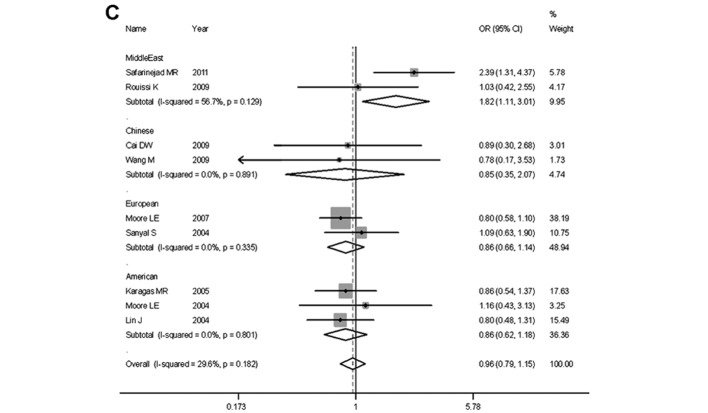
Forest plots of methylenetetrahydrofolate reductase (MTHFR) Glu429Ala polymorphism and bladder cancer risk when stratified by ethnicity. (A) Ala/Glu vs. Glu/Glu, (B) dominant model and (C) recessive model, respectively.

Overall, no significant associations of Ala222Val and Glu429Ala polymorphisms with bladder cancer risk were identified by this meta-analysis (Table III). For MTHFR Ala222Val: Val/Val vs. Ala/Ala: OR, 1.02; 95% CI: 0.80–1.29; Val/Ala vs. Ala/Ala: OR, 1.02; 95% CI: 0.92–1.12; dominant model: OR, 1.01; 95% CI: 0.87–1.17; recessive model: OR, 1.00; 95% CI: 0.87–1.15; and for MTHFR Glu429Ala: Ala/Ala vs. Glu/Glu: OR, 1.11; 95% CI: 0.78–1.58; Ala/Glu vs. Glu/Glu: OR, 1.16; 95% CI: 0.95–1.40; dominant model: OR, 1.15; 95% CI: 0.94–1.41; recessive model: OR, 0.96; 95% CI: 0.79–1.15. In the subgroup analyses by ethnicity, no significant associations were found in any of the genetic models for the MTHFR Ala222Val polymorphisms with bladder cancer risk. By contrast, the Glu429Ala polymorphism was found to be significantly associated with increased bladder cancer risk in individuals of Middle Eastern descent (Ala/Glu vs. Glu/Glu: OR, 2.11; 95% CI: 1.26–3.53; dominant model: OR, 2.16; 95% CI: 1.16–4.01; recessive model: OR, 1.82; 95% CI: 1.11–3.01), whereas no significant associations were identified in individuals of Chinese descent (Ala/Glu vs. Glu/Glu: OR, 0.98; 95% CI: 0.76–1.27; dominant model: OR, 0.97; 95% CI: 0.75–1.25; recessive model: OR, 0.85; 95% CI: 0.35–2.07), European descent (Ala/Glu vs. Glu/Glu: OR, 1.06; 95% CI: 0.91–1.24; dominant model: OR, 1.03; 95% CI: 0.89–1.20; recessive model: OR, 0.86; 95% CI: 0.66–1.14), and American descent (Ala/Glu vs. Glu/Glu: OR, 1.01; 95% CI: 0.84–1.21; dominant model: OR, 0.98; 95% CI: 0.82–1.17; recessive model: OR, 0.86; 95% CI: 0.62–1.18). The results of the subgroup analysis by ethnicity were also shown by forest plots in Fig. 1. In the subgroup analysis by the source of controls, no significant associations were found in any of the genetic models for the two polymorphisms (Table III).

### Heterogeneity and sensitivity analysis

In the heterogeneity analysis, the Val/Val vs. Ala/Ala model and dominant genetic model for the Ala222Val polymorphism, as well as the Ala/Ala vs. Glu/Glu, Ala/Glu vs. Glu/Glu and dominant genetic models for the Glu429Ala polymorphism were found to be significant (Ph<0.05 by Q test, Table III). The Ph value of the subgroup analysis showed that the heterogeneity was effectively decreased in some of the comparisons and the major source of heterogeneity may stem from the hospital-based controls and ethnicity, such as the Chinese and American subgroups. In the sensitivity analyses, with each study been excluded one at a time during the analysis, the overall results were not altered and no different conclusions were obtained, although the heterogeneity of the analysis was obviously decreased during the exclusion.

### Publication bias test

**Figure 2:**
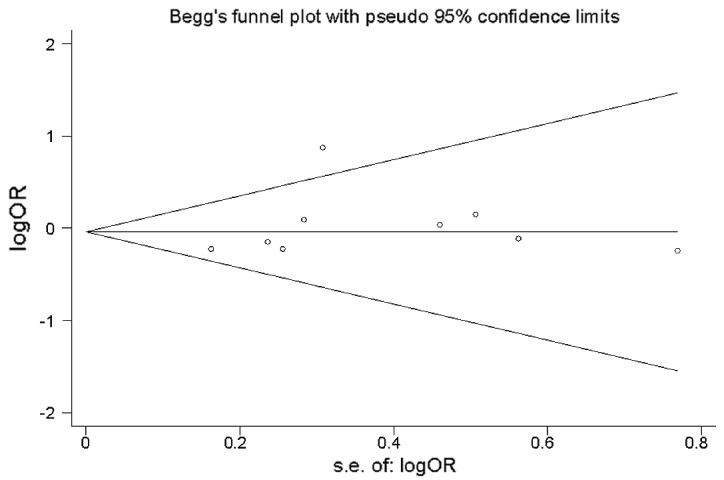
Funnel plot analysis for odds ratios (ORs). Recessive genetic model of methylenetetrahydrofolate reductase (MTHFR) Glu429Ala polymorphism in overall studies is shown as an example.

Begg’s funnel plot and Egger’s test were applied to assess the publication bias of the studies. No obvious asymmetry was identified by the Begg’s plots. The funnel plot for ORs of the recessive model for Glu429Ala (Ala/Ala vs. Ala/Glu + Glu/Glu) was shown in Fig. 2 as an example. Furthermore, the results of Egger’s test did not show any evidence of publication bias (for the Ala222Val polymorphism: P=0.961 for Val/Val vs. Ala/Ala, 0.558 for Val/Ala vs. Ala/Ala, 0.884 for the recessive genetic model and 0.810 for the dominant genetic model; for the Glu429Ala polymorphism: P=0.468 for Ala/Ala vs. Glu/Glu, 0.457 for Ala/Glu vs. Glu/Glu, 0.440 for the recessive genetic model and 0.362 for the dominant genetic model, respectively).

## Discussion

The MTHFR Ala222Val and Glu429Ala polymorphisms have been found to be a risk factor for a variety of cancers including colon cancer (11), acute lymphoblastic leukemia (12), gastric cancer (13) and head and neck squamous cell carcinoma (14). As for bladder cancer, several epidemiological studies (5–7, 18–27) have been investigated for their association with cancer risk. However, the results are not conclusive due to small sample-sized association studies that lack statistical power. In the current meta-analysis, a more precise estimation of MTHFR Ala222Val and Glu429Ala and bladder cancer was derived by including a pooled total of 3,463 cases and 3,927 controls for Ala222Val in 11 studies and 3,177 cases and 3,502 controls for Glu429Ala in 9 studies.

The data showed that the Ala222Val and Glu429Ala polymorphisms were not significantly associated with bladder cancer susceptibility in the entire population. Furthermore, the role of Ala222Val and Glu429Ala polymorphism was evaluated in different subgroups (Chinese, European, American and individuals of Middle Eastern descent), and the results indicated that the Ala222Val polymorphism was not significantly associated with bladder cancer risk in any of the genetic models. Notably, Glu429Ala polymorphism was significantly associated with bladder cancer risk in individuals of Middle Eastern descent. Individuals of Middle Eastern descent who had the Ala/Ala allele were ~82% more likely to have bladder cancer than those who had Ala/Glu or Glu/Glu genotype. Our results suggest that the Glu429Ala polymorphism was not significantly associated with bladder cancer risk in individuals of Chinese, European or American descent. The reason may be attributed to genetic and environmental factors. Since cancer is a complicated multi-genetic disease, different genetic backgrounds may contribute to the discrepancy that the same polymorphisms play different roles among different ethnic populations (28).

As a potential problem when interpreting the results of all meta-analyses (29), heterogeneity was reduced in this study. Significant between-study heterogeneity existed in the Val/Val vs. Ala/Ala and dominant genetic models of the Ala222Val polymorphism, as well as Ala/Ala vs. Glu/Glu, Ala/Glu vs. Glu/Glu and the dominant genetic model for the Glu429Ala polymorphism when all studies were pooled. Following the subgroup analyses by source of control, the heterogeneity among the Val/Val vs. Ala/Ala and dominant genetic models of the Ala222Val polymorphism arose from the hospital-based subgroup. By contrast, heterogeneity of the Ala/Ala vs. Glu/Glu, Ala/Glu vs. Glu/Glu and dominant genetic models of the Glu429Ala polymorphism arose from hospital-based subgroups, as well as from ethnicity, which was effectively decreased in the Chinese, European and American subgroup analyses. There are two reasons for the heterogeneity: i) hospital-based controls did not represent the entire population and ii) differences of genetic backgrounds and environmental factors potentially exist among different ethnical subgroups.

The associations between the Ala222Val and Glu429Ala polymorphisms and bladder cancer risk have been previously studies. For the variant genotype of Ala222Val polymorphism, Lin *et al* (5), Cai *et al* (23) and Wang *et al* (25) reported that it was associated with a higher risk of bladder cancer. Safarinejad *et al* (27) reported that the polymorphism was not associated with bladder cancer but was associated with increased risk of muscle-invasive bladder cancer. By contrast, Moore *et al* (19) reported conflicting results, suggesting a lower risk of bladder cancer was observed in individuals carrying either the Ala/Val or Val/Val genotype compared to those carrying the Ala/Ala genotype. The remaining 6 studies reported that there were no statistically significant associations between the Ala222Val polymorphism and bladder cancer risk (6–7,18,20–21,26). For the variant genotypes of Glu429Ala polymorphism, Rouissi *et al* (6) reported that it was associated with a higher risk of bladder cancer. However, Safarinejad *et al* (27) reported that it was not associated with bladder cancer but associated with increased risk of muscle-invasive bladder cancer. The remaining 7 studies reported that there were no statistically significant associations between the Glu429Ala polymorphism and bladder cancer risk (5,7,19–21,23,25).

Although Wang *et al* (25) previously published a case-control study with meta-analysis, in which the data included were up to 2007 and 7 case-control studies were analyzed with conclusions suggesting no significant associations between Ala222Val and Glu429Ala polymorphisms and bladder cancer risk, which was in accordance with results of this meta-analysis. However, in the demographic characteristics of that study (25), all the cases and controls of different studies, including individuals from the USA, Argentina and Tunis (mistaken for Turkey in that study) were considered Caucasian, rendering the subgroup analyses by ethnicity impossible, thereby discounting the statistical power of analysis for the estimation of genetic effects. In the present study, the Glu429Ala polymorphism was found to be significantly associated with increased bladder cancer risk in individuals of Middle Eastern descent.

Given the data was abstracted from these publications, there are some limitations that should be addressed with regard to this meta-analysis. Firstly, the ethnicity of the cases and controls were not uniformly defined. The Glu429Ala polymorphism for the Middle Eastern individuals, which was found to be significant, had only 2 publications with 343 cases and 507 controls in total. Secondly, the controls may not truly represent the populations, since most of the controls included in this study were hospital-based controls and the Ala222Val and Glu429Ala polymorphism is known to be potentially associated with other diseases. Thirdly, some of the environmental risk factors of bladder cancer, such as cigarette smoking habits and occupational exposure were not uniformly provided by the original studies. Furthermore, some inevitable publication bias might exist in the publications. Therefore, the results of this study should be interpreted with caution and additional studies with a large amount of data are needed for evaluation.

In conclusion, the results of the present meta-analysis suggest that heterozygous and homozygous variant carriers of the MTHFR Glu429Ala polymorphism are significantly associated with increased bladder cancer risk in individuals of Middle Eastern descent, with no evidence of an association between this polymorphism and bladder cancer risk in individuals of Chinese, European and American descent. Since the Ala/Ala genotype is relatively infrequent in the population, more well-designed studies with larger sample sizes are required to confirm the findings of the present study.

## Figures and Tables

**Table I:** Characteristics of the case-control studies considered in the meta-analysis.

Authors (refs.)	Year	Ethnicity	Genotyping method	HWE	Control source	Demographic characteristics	

						Cases	Controls
Safarinejad *et al* (27)	2011	Iranian (Asian)	PCR-RFLP	0.56	HB	158 bladder cancer patients, mean age: 62.7±10.6 years	316 controls, mean age: 61.6±9.4 years
Chung *et al* (26)	2010	Chinese-Taiwan (Asian)	PCR-RFLP	0.26	HB	150 bladder cancer patients, mean age: 65.3±1.1 years	300 controls, mean age: 66.2±0.7 years
Cai *et al* (23)	2009	Chinese (Asian)	PCR-RFLP	0.08	HB	312 bladder cancer patients, mean age: 63.1±11.3 years	325 controls, mean age: 63.7±12.2 years
Rouissi *et al* (6)	2009	Tunisian (African)	PCR-RFLP	0.49	PB	185 bladder cancer patients, mean age 67.5±9.7 years	191 controls, mean age (match the case)
Wang *et al* (25)	2009	Chinese (Asian)	PCR-RFLP	0.07	HB	239 bladder cancer patients, pack years <55 (n=42); 55–65 (77); >65 (n=120)	250 controls, pack years <55 (n=45); 55–65 (n=81); >65 (n=124)
Moore *et al* (7)	2007	Spanish (European)	TaqMan and Golden Gate	0.48	HB	1,150 bladder cancer patients, mean age: 66.0±10.0 years	1,149 controls, mean age: 65.0±10.0 years
Karagas *et al* (21)	2005	USA (American)	PCR-RFLP	0.70	PB	352 bladder cancer patients, pack years ≤40 (9); 41–55 (n=60); 56–70 (n=203); >70 (n=80)	551 controls, pack years ≤40 (31); 41–55 (n=110); 56–70 (n=304); >70 (n=106)
Moore *et al* (19)	2004	Argentina (American)	PCR-RFLP	0.29	PB	110 bladder cancer patients, mean age: 68.1 (range, 20–80 years)	110 controls, mean age: 68.4 years (match the case)
Lin *et al* (5)	2004	USA (American)	PCR-RFLP	0.07	HB	457 bladder cancer patients, mean age: 65.0 (range, 18–86 years)	457 controls, mean age: 64.0 (range, 21–89 years)
Sanyal *et al* (20)	2004	Swedish (European)	PCR-RFLP	0.82	HB	327 bladder cancer patients, mean age: 70.0 (range, 33–96 years)	246 controls, mean age (match the case)
Kimura *et al* (18)	2001	Germany (European)	PCR-RFLP	0.17	HB	165 bladder cancer patients, mean age: 67.4±11.5 years	150 controls, mean age: 62.0±11.4 years

HWE, Hardy-Weinberg equilibrium; PCR-RFLP, polymerase chain reaction-restriction fragment length polymorphism; HB, hospital-based; PB, population-based.

**Table II:** Distribution of methylenetetrahydrofolate reductase (MTHFR) gene Ala222Val and Glu429Ala genotypes for bladder cancer patients and controls.

Study (refs.)	Ethnicity	Ala222Val of MTHFR							Glu429Ala of MTHFR						
	
		Sample size (case/control)	Case			Control			Sample size (case/control)	Case			Control		
	
			AA	AV	VV	AA	AV	VV		GG	GA	AA	GG	GA	AA
Safarinejad *et al* (27)	Iranian	158/316	67	74	17	144	142	30	158/316	48	85	25	178	115	23
Chung *et al* (26)	Chinese	150/300	80	57	13	141	123	36	N/A	N/A	N/A	N/A	N/A	N/A	N/A
Cai *et al* (23)	Chinese	312/325	82	169	61	113	170	42	312/325	215	91	6	226	92	7
Rouissi *et al* (6)	Tunisian	185/191	87	86	12	81	90	20	185/191	97	78	10	121	60	10
Wang *et al* (25)	Chinese	239/250	66	128	45	88	132	30	239/250	169	67	3	171	75	4
Moore *et al* (7)	Spanish	1,041/1,049	418	478	145	402	486	161	1,068/1,078	537	457	74	557	429	92
Karagas *et al* (21)	American	350/543	140	171	39	227	245	71	350/542	173	146	31	267	220	55
Moore *et al* (19)	Argentina	106/109	45	42	19	32	59	18	106/108	52	45	9	55	45	8
Lin *et al* (5)	American	448/448	199	197	52	218	177	53	448/447	219	199	30	213	197	37
Sanyal *et al* (20)	Swedish	309/246	173	113	23	121	102	23	311/245	145	133	33	110	111	24
Kimura *et al* (18)	Germany	165/150	70	80	15	65	73	12	N/A	N/A	N/A	N/A	N/A	N/A	N/A

AA, Ala/Ala; AV, Ala/Val; VV, Val/Val; GG, Glu/Glu; GA, Glu/Ala; N/A, not available.

**Table III:** Results of meta-analysis for methylenetetrahydrofolate reductase (MTHFR) gene Ala222Val and Glu429Ala polymorphism and bladder cancer risk.

Genetic model		Recessive model		Dominant model		Homozygote		Heterozygote	
				
Ala222Val	No. of study (sample size case/control)	Val/Val vs. Val/Ala + Ala/Ala		Val/Val + Val/Ala vs. Ala/Ala		Val/Val vs. Ala/Ala		Val/Ala vs. Ala/Ala	
			
		OR (95% CI)	Ph	OR (95% CI)	Ph	OR (95% CI)	Ph	OR (95% CI)	Ph
Overall	11 (3,463/3,927)	1.00 (0.87–1.15)	0.130	1.01 (0.87–1.17)	0.024	1.02 (0.80–1.29)	0.019	1.02 (0.92–1.12)	0.103
Ethnicity									
Chinese	3 (701/875)	1.32 (0.80–2.15)	0.070	1.19 (0.79–1.78)	0.029	1.42 (0.72–2.78)	0.016	1.16 (0.93–1.45)	0.146
European	3 (1,515/1,445)	0.89 (0.72–1.11)	0.747	0.90 (0.78–1.05)	0.485	0.86 (0.68–1.09)	0.629	0.92 (0.79–1.07)	0.558
American	3 (904/1,100)	0.93 (0.71–1.22)	0.759	0.98 (0.71–1.36)	0.063	0.95 (0.71–1.26)	0.690	0.97 (0.66–1.43)	0.031
Middle East	2 (343/507)	0.87 (0.54–1.40)	0.184	0.98 (0.74–1.30)	0.270	0.87 (0.53–1.44)	0.134	1.00 (0.75–1.34)	0.440
Source of control									
PB	3 (641/843)	0.83 (0.60–1.14)	0.496	0.92 (0.74–1.13)	0.109	0.78 (0.56–1.11)	0.589	0.86 (0.57–1.29)	0.057
HB	8 (2,822/3,084)	1.08 (0.86–1.35)	0.099	1.06 (0.89–1.25)	0.033	1.12 (0.83–1.50)	0.013	1.03 (0.92–1.15)	0.203

Glu429Ala	No. of study (sample size case/control)	Ala/Ala vs. Ala/Glu + Glu/Glu		Ala/Ala + Ala/Glu vs. Glu/Glu		Ala/Ala vs. Glu/Glu		Ala/Glu vs. Glu/Glu	
			
		OR (95% CI)	Ph	OR (95% CI)	Ph	OR (95% CI)	Ph	OR (95% CI)	Ph

Overall	9 (3,177/3,257)	0.96 (0.79–1.15)	0.182	1.15 (0.94–1.41)	<0.001	1.11 (0.78–1.58)	0.009	1.16 (0.95–1.40)	0.002
Ethnicity									
Chinese	2 (551/575)	0.85 (0.35–2.07)	0.891	0.97 (0.75–1.25)	0.595	0.85 (0.35–2.07)	0.857	0.98 (0.76–1.27)	0.599
European	2 (1,379/1,078)	0.86 (0.66–1.14)	0.335	1.03 (0.89–1.20)	0.515	0.88 (0.66–1.17)	0.512	1.06 (0.91–1.24)	0.333
American	3 (904/1,097)	0.86 (0.62–1.18)	0.801	0.98 (0.82–1.17)	0.915	0.86 (0.62–1.20)	0.781	1.01 (0.84–1.21)	0.963
Middle East	2 (343/507)	1.82 (1.11–3.01)	0.129	2.16 (1.16–4.01)	0.032	2.35 (0.75–7.40)	0.040	2.11 (1.26–3.53)	0.089
Source of control									
PB	3 (641/841)	0.93 (0.64–1.36)	0.839	1.13 (0.92–1.39)	0.188	0.97 (0.66–1.44)	0.726	1.16 (0.93–1.44)	0.204
HB	6 (2,536/2,416)	1.04 (0.72–1.51)	0.052	1.15 (0.87–1.52)	<0.001	1.15 (0.68–1.93)	0.001	1.14 (0.89–1.48)	0.001

OR, odds ratio; CI, confidence interval; Ph, P-values for heterogeneity from Q test; PB, population-based; HB, hospital-based. Random-effects model was used when Ph<0.05; otherwise, fixed-model was used.
